# Supplementary material for: Platyphylloside Isolated from Betula platyphylla is Antiproliferative and Induces Apoptosis in Colon Cancer and Leukemic Cells
Source: Molecules. 2019 Aug 15;24(16):2960. doi: 10.3390/molecules24162960 (PMC6720625; doi:10.3390/molecules24162960)
Supplement: Supplementary file 1 [file molecules-24-02960-s001.pdf]

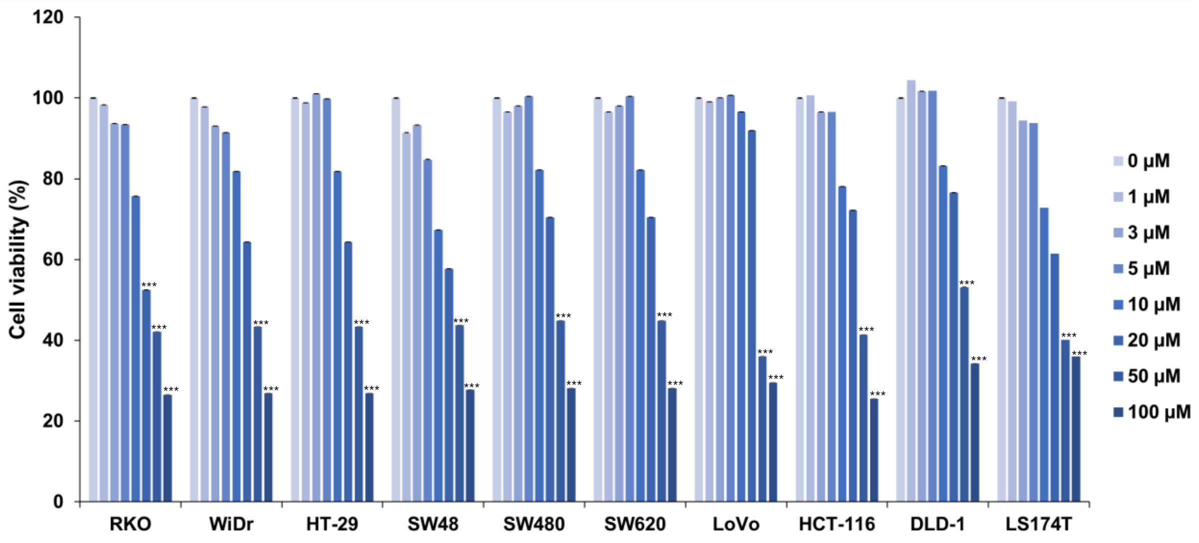

Figure S1. BPP showed anti-proliferative effect on various colon cancer cells. Cell viability was determined using the MTS assay post treatment with compound.

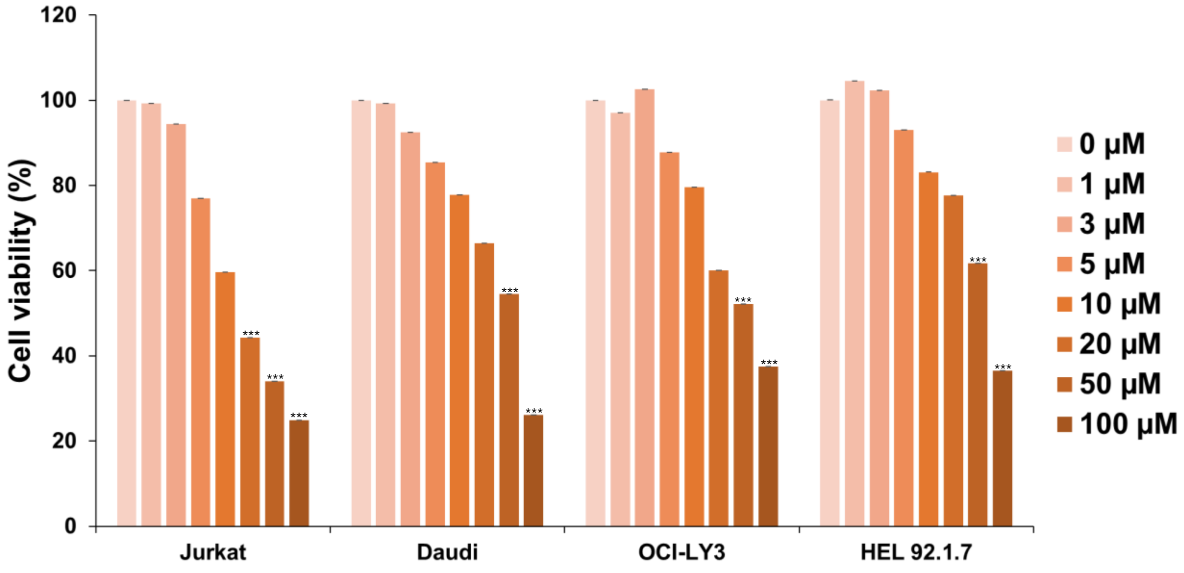

Figure S2. BPP showed anti-proliferative effect on various leukemia cells. Cell viability was determined using the MTS assay post treatment with compound.
